# Cincinnati Dental Association

**Published:** 1858-11

**Authors:** 

**Affiliations:** Cincinnati, Ohio


### CINCINNATI DENTAL ASSOCIATION.

CINCINNATI, Ohio, October, 1858.

FRIEND TOLAND:—We have the pleasure of announcing to our professional brethren at the different quarters of the globe and all intermediate points, that there is now a *local* dental association within our midst; yea, even in the city of Cincinnati itself. Truly, the late "*huge*" convention did arouse the latent energies of this locality, and it has manifested itself in the production of a society whose prospects far surpass the most sanguine expectations of its strongest advocates and prime movers.

The preliminary meeting was held at the office of our progressive friends, Drs. Hamlen & Smith, and resulted in the appointment of a committee to draft a constitution and by-laws for the government and support of the future structure. Dr. Bonsall officiated as chairman. Much enthusiasm prevailed, and some *patriotic* speeches were "done." The committee on constitution only reported partially, the first meeting, received instructions and were requested to report in full at the next meeting, which was held at the office of our magnanimous brother, Dr. S. Wardle, on the following Wednesday evening.

The second meeting was called to order, promptly, by the chairman, and the minutes of the former meeting read and, *of course*, received. The "constitutional" committee made a report of a constitution only, which is an admirable production, and reflects much credit upon them. By-laws were not reported, but, I presume, will be, sometime (that is) soon. The election for permanent officers resulted as follows: *President*, Dr. C. Bonsall; *Vice President*, Dr. H. R. Smith; *Recording Secretary*, Dr. C. H. James; *Corresponding Secretary*, Dr. J. Taft; *Treasurer*, Dr. Wheeler. So you see, under the guidance of such excellent officers, it must *go on*.

The third meeting was postponed, in consequence of an important State and county election, which happened upon the same day the society met; however, the enthusiasm still prevails, and we may look for an eloquent discussion of the "Electro-Magnetic" question, at the next meeting, which will transpire sometime next month.

The following doctors are members of the Society: Dr. Jas. Taylor, Dr. C. Bonsall, Drs. Hamlen & Smith, Dr. J. T. Irwin, Dr. Wheeler, Dr. Richardson, Dr. S. Wardle, and Dr. C. H. James. I suppose other names will soon be added to the list.

(In haste), JUNIUS.

